# Optimization of the object recognition assay to test mouse cognition

**DOI:** 10.1002/alz.093258

**Published:** 2025-01-03

**Authors:** Kumar Sambamurti, Dallas Crowder, Miguel A Pappolla, Suhua Shah, Hongkuan Fan, Debomoy K. Lahiri, Nigel H Greig, Anumantha Kanthasamy

**Affiliations:** ^1^ Medical University of South Carolina, Department of Neurosciences, Charleston, SC USA; ^2^ Medical University of South Carolina, Charleston, SC USA; ^3^ University of Texas Medical Branch, Galveston, TX USA; ^4^ Indiana Alzheimer’s Disease Research Center, Indianapolis, IN USA; ^5^ National Institute on Aging, NIH, Baltimore, MD USA; ^6^ University of Georgia, Athens, GA USA

## Abstract

**Background:**

Alzheimer’s disease (AD) has traditionally been recognized as progressive dementia with brain deposits of amyloid (Aβ) and Tau (MAPT) proteins starting 20 and 10 years before the onset of clinical symptoms. Aggregation and deposition of Aβ and Tau proteins have been successfully studied in vitro, cell cultures, and animal models, but clinical deficits have been more difficult to assess. Behavior in mice is a complex phenomenon and subject to variation based on mouse interest, moods, stress‐induced distraction, and other undefined parameters. We have attempted to optimize the novel and displaced object assays to improve behavior studies to evaluate animal models of neurodegeneration.

**Method:**

5xFAD, hTau, and related mice were habituated in the field for 15 minutes twice on day‐1, followed by familiarization with two identical objects twice for 15 minutes on day‐2. The final testing on day‐3 started with novel followed by displaced object analysis. We compared three opaque arenas –rectangular (34 × 24 cm), large square (40 cm), and small square (25 cm) and objects of several heights and types. We also assessed handling mice by cupping to make them more interactive and relaxed for the study.

**Result:**

Mice tend to jump over the arena walls and escape when handled by their tail but are contained when handled by cupping through a cylinder. All the systems were equally able to discriminate between novel and familiar objects. However, we could only distinguish between the familiar and displaced objects in the smaller 25 cm square arena. Also, object interaction time was longer in the smaller field.

**Conclusion:**

It is critical to handle mice by cupping to ensure reduced stress and optimum object interaction. A smaller arena may promote object interaction; taller objects reduce detection artifacts. NIH grants supported the study.

